# Double-Walled Poly-(D,L-lactide-co-glycolide) (PLGA) and Poly(L-lactide) (PLLA) Nanoparticles for the Sustained Release of Doxorubicin

**DOI:** 10.3390/polym13193230

**Published:** 2021-09-23

**Authors:** M. Margarida Cardoso, Inês N. Peca, Telma Lopes, Rui Gardner, A. Bicho

**Affiliations:** 1 LAQV-REQUIMTE, Departamento de Química, Faculdade de Ciências e Tecnologia, Universidade NOVA de Lisboa, Quinta da Torre, 2829-516 Caparica, Portugal; nobre.iines@gmail.com; 2 Instituto Gulbenkian de Ciência, Rua da Quinta Grande 6, 2780-156 Oeiras, Portugal; telma.lopes@unibas.ch (T.L.); gardner@mskcc.org (R.G.); a.bicho@dq.fct.unl.pt (A.B.)

**Keywords:** drug delivery, PLGA/PLLA, double-walled nanoparticles, controlled release, doxorubicin

## Abstract

Double-walled nanoparticles (DWNPs), containing doxorubicin as a model drug, were produced using poly-(D,L-lactide-co-glycolide) (PLGA) and poly(L-lactide) (PLLA) by the solvent evaporation technique. Double-walled microparticles containing doxorubicin were also produced to make possible the examination of the inner morphology and drug distribution using optical and fluorescence microscopy. The produced microparticles present a double-walled structure with doxorubicin solubilized in the PLGA-rich phase. The DWNPs produced present very low initial burst values and a sustained DOX release for at least 90 days with release rates decreasing with the increase in the PLLA amount. Zero-order release kinetics were obtained after day 15. The results support that the PLLA layer acts as a rate control barrier and that the diffusion of doxorubicin from the drug-loaded inner PLGA core can be retarded by an increase in the thickness of the unloaded outer layer. The unloaded double-walled nanoparticles produced were used in in vitro tests with CHO cells and demonstrate that they are nontoxic, while the double-walled nanoparticles loaded with doxorubicin caused a great cellular viability and decreased when tested in vitro.

## 1. Introduction

The use of nanoparticles (NP) as drug carriers is one of the most promising areas of human health care science since they can circulate in the blood or cross cell membranes and be internalized [1,2,3,4,5,6]. Drugs are frequently limited by dose-limiting toxicity and, therefore, it is important to control their release during the transportation to the site of action. NP are an excellent system for this matter, since it is possible to easily manipulate their size and surface characteristics, therefore, altering their behavior towards a desired goal. Controlled release and particle degradation characteristics can be readily modulated by the choice of matrix constituents and the concentration of loaded drug. Most of the studies published so far were performed with a single-polymer layer NP where a high initial burst release generally occurs, which present a limitation in clinical application. This restriction can be overcome using particles containing two immiscible polymers that form a core (internal) and a shell (outer) layer, commonly known as double-walled (DW) particles. Furthermore, the use of DW particles allows the drug release rate to be tuned by choosing the suitable core and shell polymers and adjusting the thickness of the outer layer by handling the polymers mass ratio. This approach provides more options concerning the type of drug release profiles intended for each type of treatment [7]. Matsumoto and co-workers found that the phenomenon of polymer-polymer phase separation can be applied with the Poly(L-lactide) (PLLA)/Poly(DL-lactide-co-glycolide) (PLGA) system [8], both biodegradable and biocompatible polymers that have generated tremendous interest [9,10]. Studies using this system and copolymers of PLGA to produce double-walled microparticles (DWMP) for the sustained release of doxorubicin [11], etanidazole [12,13,14], 5-fluorouracil [15] and globular proteins [16,17,18] have been reported. In these systems, it was found that the drug is distributed in the inner core material and the shell can act as a rate-limiting barrier to drug release. Our group previously reported the production of double-walled nanoparticles (DWNPs) containing meloxicam using PLLA/PLGA that can ally the benefits of a double-layer system to those of NP [19]. While studies of drug release from double-layered particles produced with different mass polymer ratios have been reported for microparticles, no such studies have been conducted using nanoparticles. Therefore, the aim of the present study is to produce drug-loaded DWNPs using PLLA and PLGA polymers with different mass ratios to test for the possibility to achieve slower drug releases and lower initial burst releases when compared to one-layer systems

The model drug chosen for this work is a water-soluble drug, doxorubicin (DOX), an anthracycline used in the treatment of a wide range of cancers, including many types of carcinomas (solid tumors). It functions by inhibiting nucleic acids synthesis within cancer cells, but it is rapidly cleared from the blood and leads to various dose-limiting side effects [20,21]. DWNPs were characterized in terms of morphology, size, zeta potential and crystallinity and further used for in vitro release studies to evaluate whether a PLLA layer surrounding a PLGA core can be used to adjust the drug release rate and for in vitro assays in mammalian cell cultures to assess their therapeutic potential.

## 2. Experimental

### 2.1. Materials

PLLA (Mw 85,000–160,000), PLGA (50:50) (Mw 40,000–75,000), Poly(vinyl alcohol) (PVA) (Mw 30,000–70,000; degree of hydrolysis: 87–90%) used as the emulsifying agent and doxorubicin hydrochloride were obtained from Sigma Aldrich (Saint-Louis, MO, USA). Dichloromethane (DCM), (99.9% purity), acetone (95% purity) and phosphate-buffered saline (PBS) were purchased from Fluka (Buchs, Switzerland). Culture media, serum, antibiotics and fungizone and fetal bovine trypsin, were purchased from Gibco (Grand Island, NY, USA). All other materials or solvents used were of analytical grade. Live/Dead viability kit and Toto-3 were achieved from Invitrogen (Grand Island, NY, USA).

### 2.2. Preparation of PLLA/PLGA Double-Walled Nanoparticles and PLGA Nanoparticles

PLLA/PLGA double-walled nanoparticles were prepared, utilizing the polymer incompatibility between PLLA and PLGA which result in their complete phase separation [8], using the oil-in-water-in water emulsion solvent extraction/evaporation technique according to Albuquerque et al. [19]. Briefly, PLLA and PLGA were separately dissolved in DCM in a polymer mass ratio of PLLA to PLGA of 2:1, 4:1 and 6:1 *w/w* using a total polymer quantity of 200 mg and a total DCM volume of 1 mL. The two polymeric solutions were mixed in the vortex and subsequently added to 10 mL of an aqueous solution containing PVA 3% *w/v* as a stabilizer and sonicated for 30 s at 100 W (Model UP100H, MS2, Hielscher) to produce the first o/w emulsion. The formed o/w emulsion was poured dropwise into 300 mL of a 0.25% (*w/v*) PVA aqueous solution and magnetically stirred for 24 h at room temperature to extract/ evaporate the organic solvent and harden the particles. The produced NP were collected by centrifugation at 13,257× *g* for 10 min (Sartorius, Sigma 4K15), washed three times with deionised water and freeze dried (Telstar Cryodos).

DWNPs containing DOX were prepared adding 10 mg of DOX to the polymer solution and using the same procedure. PLGA NP-containing DOX were prepared in the same manner using 200 mg of PLGA.

### 2.3. Preparation of PLLA/PLGA Double-Walled Microparticles

Double-walled microparticles were intentionally produced large enough to enable the observation of the DOX solubilization local by microscopy. DWMPs of PLLA/PLGA mass ratio of 2:1 were prepared using the solvent extraction/evaporation technique. A total polymer mass of 600 mg of PLLA and PLGA was separately dissolved in DCM in order to get a mass ratio of these polymers of 2:1, using a total DCM volume of 3 mL. The two polymeric solutions were mixed and 10 mg of DOX were added to this solution. This polymeric solution was added to 800 mL of an aqueous solution of PVA 0.25% (*w/v*), therefore creating an *o/w* emulsion, and stirred at a 1200 rpm rate for 2 h to allow DCM evaporation and MP hardening. The produced MP were collected by centrifugation (Sartorius, Sigma 4K15, St. Louis, MO, USA), washed with deionized water and freeze-dried (Telstar Cryodos, Mumbai, India). To identify the double-walled structure as well as the distribution of DOX in the particle, MP were incorporated in a resin, cross sectioned and their surface morphology and internal structure was observed by microscopy. MP images were taken by a digital camera Nikon DXM1200F coupled to a Zeiss Axioplan 2 Optical Microscope under a reflected-polarized normal light that allows the identification of distinct polymeric layers. Since DOX is colored, the site of solubilization of DOX within the loaded MP was also determined.

### 2.4. Characterization of NP

#### 2.4.1. Surface Morphology

The characterization of NP morphology and estimation of NP size was performed by Scanning Electron Microscopy (SEM). Images were obtained with a scanning electron microscope (model DSM 962, Zeiss, Karlsruhe, Germany) with an accelerating voltage of 20 kV after gold-coating the samples fixed onto metallic studs under argon atmosphere.

#### 2.4.2. Particles Size and Zeta Potential Analysis

The size and the zeta potential of the produced NP were measured by dynamic light scattering (DLS) performed using a Laser Light Scattering Instrument (Zetasizer Nano ZS, Malvern). Freeze-dried NP were dispersed in a PVA 0.5% (*w/v*) aqueous solution, sonicated, washed three times with deionised water and resuspended in water before each measurement. All measurements were made in triplicate.

#### 2.4.3. Thermal Analysis by Differential Scanning Calorimetry (DSC)

Thermal analysis of NP was performed using a differential scanning calorimeter (Setaram DSC131) equipped with a thermal analysis data system connected to a cooling system. Sealed aluminum pans containing 10 mg of sample were heated from −20 °C to 80 °C, cooled to 25 °C, and reheated to 250 °C at a rate of 10 °C/min under nitrogen atmosphere.

#### 2.4.4. Drug Encapsulation Efficiency (EE)

The amount of DOX encapsulated within the particles was determined by High Performance Liquid Chromatography (HPLC) (Hitachi-Merck, Okinawa, Japan). Then, 5 mg of particles were completely dissolved in 5 mL of DCM. The solution was then placed in a desiccator and after complete evaporation of DCM, 2 mL of acetonitrile: acetate buffer (pH 4) 50:50 (*v/v/v*) were added. The sample was then analyzed for its drug concentration using a Merck RP-18 (Merck, Darmstadt, Germany) column eluted with a solution acetonitrile:acetate buffer (pH 4) 50:50 (*v/v*) at a flow rate of 0.8 mL min^−1^. A UV detector (Merck Hitachi, Okinawa, Japan) at a wavelength of 234 nm was used. The encapsulation efficiency was determined as the mass ratio between the DOX amount within particles and the quantity of DOX used in the preparation.

### 2.5. In Vitro DOX Release Studies

In vitro release of DOX from DWNPs and PLGA NP was conducted dispersing 10 mg of NP in 7 mL of phosphate buffer media (PBS) at pH 7.2 in closed centrifuge tubes. The tubes were placed at a temperature of 37 °C and a speed rotation of 120 rpm in a horizontal shaker. At time intervals, the tubes were removed and centrifuged at 9000 rpm for 15 min. The supernatants were collected and the amount of DOX released was quantified as described in Section 2.4.4. The precipitated NP were re-suspended in 7 mL of fresh buffer and placed back in the incubator.

### 2.6. In Vitro Cell Assays

#### 2.6.1. Cell Culture

CHO cells (Hamster Chinese Ovary, ECACC No. 85050302) were routinely propagated in static conditions. Briefly, D-MEM culture medium (Dulbecco’s Modified Eagle Medium, Gibco) with 4.5 g/L glucose and GlutaMAX™ and without pyruvate was supplemented with penicillin and streptomycin, both 50 I.U/mL, and 10% (*v/v*) FBS. Sub-culture was performed by trypsinization when cellular growth reached approximately 70% confluence. Cultures were grown in solid supports at 37 °C in a humidified atmosphere with 5% CO_2_.

#### 2.6.2. Cell Viability Assays

The effect of unloaded NP and DOX-loaded NP on cell viability was investigated with a culture of CHO cells by counting the absolute number of cells, by flow cytometry and by fluorescence microscopy using the Live/Dead viability kit. The experiments were performed using PLGA NP and DWNPs in a 6:1 mass ratio. To prevent cell culture contaminations, NP were resuspended and washed in sterile PBS supplemented with penicillin, streptomycin and fungizone (25 µg/mL). This suspension was kept up to 2 weeks at 4 °C.

- Flow Cytometry

CHO cells were seeded in D-MEM culture medium for 24 h in a CO_2_ incubator (5% CO_2_ at 37 °C). After that, 100 μL of a DOX-loaded NP or unloaded NP suspension with a concentration of 1 mg NP/mL were added to 300 μL of cell culture. An assay with no addition of NP was also performed as a control. After 48 h, cells were collected and resuspended in 1 mL of medium. For each sample, a calcein assay (identifying live cells), a Toto-3 assay (identifying dead cells), an assay with both calcein and Toto-3 and an assay with no addition were performed. Calcein was incubated at 0.2 µM for 30 min, whereas Toto-3 was incubated for 10 min at 750 μM, both at room temperature. Flow cytometry was performed on a CyAn ADP (Beckman Coulter, Fort Collins, CO, USA). Then, 488 nm and 642 nm lasers were used to excite calcein and Toto-3, respectively. Calcein and Toto-3 were detected with a 530/30 and 665/20 nm bandpass filter, respectively.

- Absolute number of cells

CHO cells were seeded in DMEM culture medium for 24 h in a CO_2_ incubator (5% CO_2_ at 37 °C). After that, 100 μL of a DOX-loaded NP or unloaded NP suspension with a concentration of 1 mg NP/mL were added to 300 μL of cell culture. An assay with no addition of NP was also performed as a control. After 48 h, cells were collected and resuspended in 0.5 mL of medium and counted using a Scepter™ 2.0 automated cell counter (Merck Millipore, Burlington, MA, USA) with the 40 μm sensor. The results were analyzed using the Scepter™ software.

- Fluorescence microscopy

Cell viability was also analyzed by fluorescence microscopy using the Live/Dead viability kit. Briefly, 100 μL of combined LIVE/DEAD assay reagents was added to CHO cells grown in 12 mm diameter coverslips. Cells were incubated with the reagent at 37 °C for 35 min. Fluorescent intensity was measured with an excitation wavelength of 550 nm and an emission wavelength of 590 nm under the fluorescence microscope (TE2000, Nikon) equipped with a Hamamatsu Flash 2.8 chamber and Prior Lumen 200 Pro lighting. Green fluorescence (caused by calcein acetoxymethyl) indicates viable cells and red fluorescence (caused by ethidium homodimer) indicates dead cells. ImageJ was used to analyze the obtained images.

## 3. Results and Discussion

### 3.1. Nanoparticles Characterization

**Figure 1:**
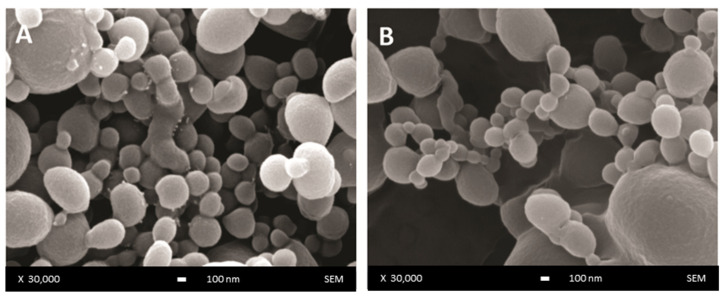
SEM images of DWNP: (**A**) PLLA/PLGA 2:1, (**B**) DOX-loaded PLLA/PLGA 2:1.

The surface morphology of unloaded and DOX-loaded DWNPs produced with 2:1, 4:1 and 6:1 PLLA: PLGA mass ratios was examined by scanning electron microscopy and representative images are presented in Figure 1. NP show a regular spherical shape and a smooth surface (Figure 1A) that is not affected by the presence of DOX (Figure 1B).

**Figure 2:**
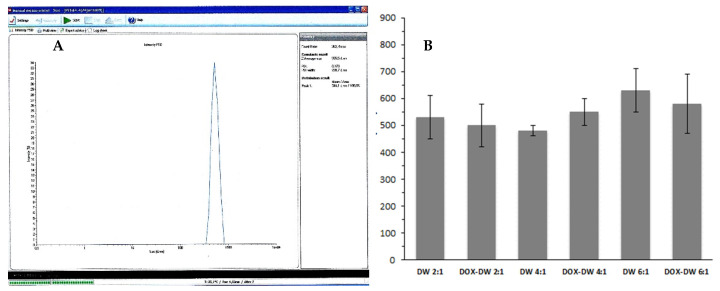
(**A**) Representative DLS result (obtained for DOX–DWNP 6:1); (**B**) Sizes of unloaded and DOX-loaded PLLA/PLGA DWNPs with mass ratios of 2:1, 4:1 and 6:1 (*n* = 3) determined by DLS.

The mean particles diameter of the produced DWNPs obtained from DLS measurements are shown in Figure 2. The sizes obtained by DLS ranges 450–610 nm for 2:1, 430–480 nm for 4:1 and 510–730 nm for 6:1 for unloaded DWNPs and 400–580 nm for 2:1, 500–600 nm for 4:1 and 450–730 nm for 6:1 for DOX-loaded DWNPs.

**Figure 3:**
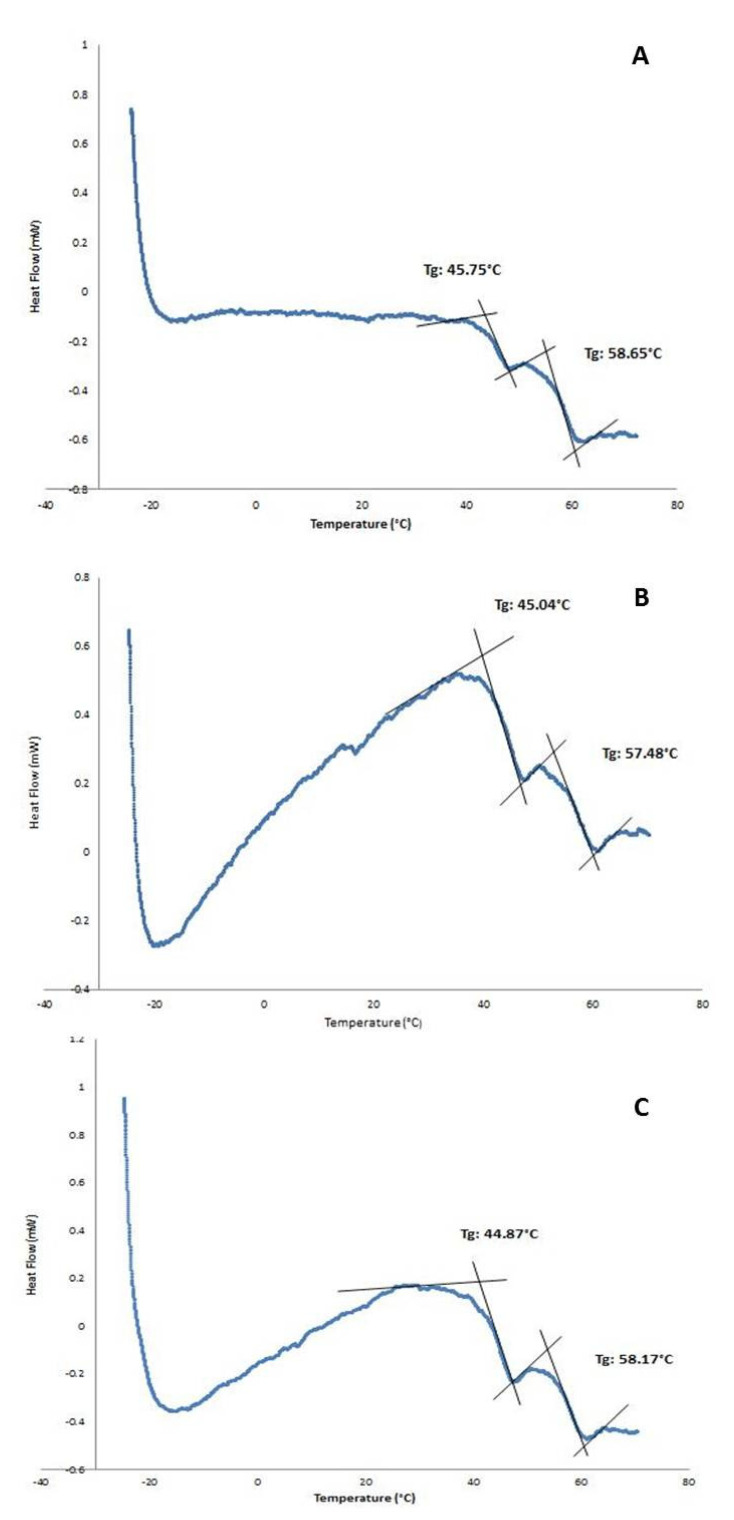
DSC thermograms of PLLA/PLGA DWNPs with mass ratios: (**A**) 2:1, (**B**) 4:1, (**C**) 6:1.

**Figure 4:**
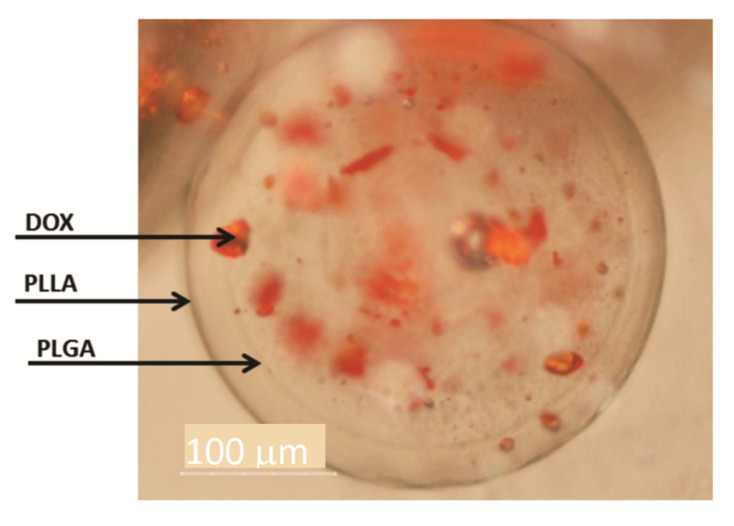
Optical microscopy of DWMP of PLLA/PLGA 2:1. The arrows indicate the outer surface, the interface between PLLA and PLGA and DOX.

The existence of a double-walled structure can be observed in the DWNPs thermograms (Figure 3), where two glass transition phenomena are evident at 44.9–45.75 °C and at 56.4–58.65 °C, near the glass transition temperatures of PLGA and PLLA, respectively, indicating that phase separation occurred. For a miscible blend, we would observe a single Tg between the Tg´s of the original polymers [22,23]. The glass transition temperatures (Tg) of the original polymers PLGA and PLLA measured were 43.0 °C and 52.1 °C for PLGA and PLLA, respectively. At 175.3 °C, one crystalline melting point was observed, attributed to PLLA, once PLGA is an amorphous polymer. In order to investigate the distribution of DOX inside PLLA/PLGA NP and since NP are too small to allow cross-sectioning, double-walled microparticles loaded with DOX were also produced, subjected to cross-sectioning and observed using an optical microscope (Figure 4). This technique allows an easy identification due to the red color of doxorubicin.

In Figure 4 a shell surrounding a core may be seen confirming the formation of double-walled microparticles where a polymer engulfs the other in a single step with the solvent extraction/evaporation technique. PLLA/PLGA DWMP with a polymer mass ratio of 2:1 produce a PLGA-rich inner phase and a PLLA-rich outer phase as concluded by Albuquerque et al. [19] and other authors [8,12]. Figure 4 also shows the preferential DOX distribution in the core, therefore, indicating that DOX has a greater affinity with PLGA. Other groups have also verified a preferential molecule distribution between PLLA and PLGA in DW microspheres. A similar distribution was obtained by Tan and Wang and Lee et al. [7,13,14] that observe that DOX solubilisation local was mainly within the PLGA-rich phase while Rahman and Mathiowitz reported that BSA was mainly located in the PLLA-rich phase [16].

The obtained DWMP can be considered a reservoir-dispersed matrix system with DOX solubilized in the PLGA core and the PLLA-rich shell acting as a barrier that can control drug release. The zeta potential of DWNPs, measured at pH 7, were −13.4 ± 0.7 mV, −12.4 ± 0.9 mV and −12.7 ± 0.4 mV, for DOX-loaded DWNPs 2:1, 4:1 and 6:1, respectively, and −13.9 ± 0.7 mV, −12.9 ± 0.9 mV and −11.1 ± 1.4 mV, for 2:1, 4:1 and 6:1 for unloaded DWNPs, respectively, indicating the presence of deprotonated PLGA carboxylic groups on the outer surface of NP. These values show that NP have colloidal stability and that the presence of DOX do not change NP superficial charge and the way these particles form clusters as DOX is loaded inside the NP.

Regarding the EE for the particles produced, defined as the percentage of the mass of DOX into NP relative to the amount of DOX used in DWNP preparation, a decrease was observed when the PLLA/PLGA mass ratio goes from 2:1 (25.6%) to 4:1 (16.3%) and to 6:1 (13.5%). This behavior can be explained by the fact that increasing the PLLA/PLGA ratio and maintaining the total polymer mass, a smaller core volume composed of PLGA to which DOX has affinity is available for DOX solubilization and therefore some DOX might be solubilized in the outer PLLA layer and can thus be lost in washing procedures during the NP production process. The EE values obtained are lower than those reported for DW microparticles, in the range between 31% and 87% [7,8,15,17]. This lower EE values can be attributed to a larger loss of drug to the aqueous phase that can occur during the first emulsification process when high sonication powers are applied. The EE obtained for DOX (25.6%) in a PLLA/PLGA mass ratio 2:1 is similar to that reported for meloxicam-loaded DWNPs with a PLLA/PLGA mass ratio of 1:1 (26%) [19].

### 3.2. In Vitro Controlled Drug Release

**Figure 5:**
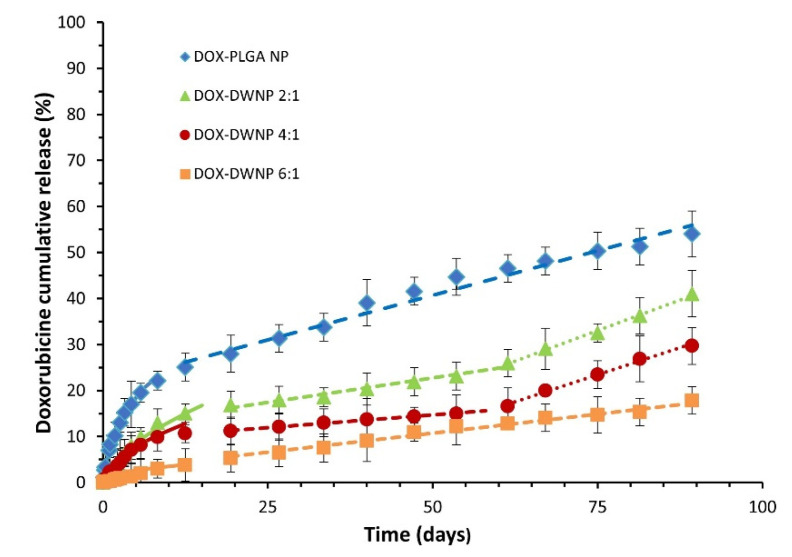
Cumulative DOX release profiles from PLGA NP and DWNPs made of PLLA and PLLG in the mass ratios of 2:1, 4:1 and 6:1, expressed as the percentage of DOX released. Solid lines were obtained by the Korsmeyer Peppas model (Equation (1)). Dashed lines obtained by zero order kinetic models.

To compare the release profile of doxorubicin from DWNPs with different mass ratios of PLLA/PLGA and from monolithic PLGA NP, drug release tests were performed under the same conditions (Figure 5). The cumulative in vitro release of DOX from DWNPs with PLLA as the shell material and PLGA as the core material shows different profiles than those obtained with monolithic PLGA NP, with much lower initial burst release values and slower drug release rates. They are characterized by a very low initial burst release, assumed to result from the diffusion of the drug located on or near the outer surface of the DWNPs, followed by a sustained release of doxorubicin for 96 days during which different rates depending on the release mechanisms involved [24] can be observed.

The obtained profiles indicate that the largest amount of doxorubicin was encapsulated within the polymeric matrix rather than adsorbed in the surface of NP. In order to evaluate the weight of Fickian and non-Fickian behaviors on the overall drug delivery [25], the release data obtained were fitted to a semi-empirical Korsmeyer-Peppas model equation (Equation (1)):

(1) $$\frac{M_{t}}{M_{\inf}}=kt^{n},$$

where *M_t_*/*M*_inf_ is the fraction of drug released at time *t*, *k* is a kinetic constant characteristic of the network structure and *n* is a diffusional exponent that indicates the mechanism of transport of a drug through the polymeric matrix. The parameters *n* and *k* were obtained fitting the experimental drug release data for *M_t_* < 60% to Equation (1) using the simplex algorithms and the least squares minimization of the residuals. The results, within 95% confidence level, are presented in Table 1. The Korsmeyer-Peppas model describes well experimental values obtained until about day 15 for all DWNPs, with *n* values ranging from 0.43 to 0.58, which indicates a strong contribution of Fickian diffusion in DOX release process. The value of *n* presented by DWNP 2:1 (0.58) is in the anomalous transport range (0.43 < *n* < 0.85) and indicates that, despite the main contribution of diffusion to the transport, some polymer chains relaxation might be present. This value is the closest to the value obtained by PLGA NP (0.62), as expected. It can be observed that the value of *n* decreases with an increase in the PLLA amount in the outer shell achieving 0.43, typical of a pure Fickian transport, for DWNP 6:1. This behavior can be explained by the lower chain flexibility of PLLA polymer (that has a higher Tg) when compared with PLGA. After the 15th day, all NP present a zero-order release kinetic that can be due to particles erosion and polymers degradation. During this phase and until 2 months DWNPs 2:1 and 4:1 show a zero-order kinetic with rates of 0.19% and 0.12% DOX release day^−1^, respectively, much lower than that obtained for PLGA NP (0.43% DOX release day^−1^).

After 2 months, the release rates increase to 0.48% and 0.44% DOX release day^−1^, similar to the value determined for PLGA. These results support that PLLA acts as a rate-limiting barrier and strongly indicate that a non-drug-loaded outer layer can be effectively used to adjust the drug release rate of DOX from the drug-loaded inner PLGA core, therefore, allowing a zero-order kinetic release to be achieved. There are no published studies concerning drug release from double-walled nanoparticles. However, similar profiles, exhibiting a low initial burst release followed by a characteristic lag phase and an almost linear sustained release up to 2 months, have been obtained for double-walled microparticles composed of PLLA/PLGA and PLGA(80/20)/PLGA(75/25) DWMP [7,12,15,24].

### 3.3. Viability Assays

**Figure 6:**
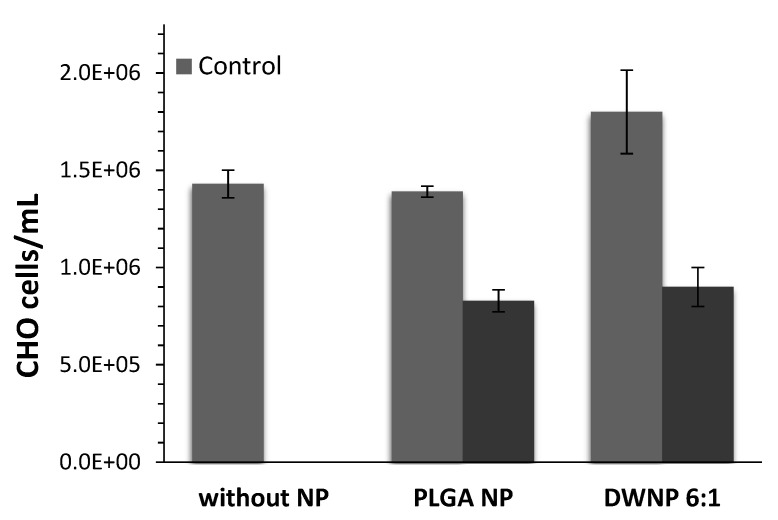
Absolute number of CHO cells in the presence of 1 mg/mL of unloaded or doxorubicin-loaded PLGA NP and DWNPs 6:1, (*n* = 3).

**Figure 7:**
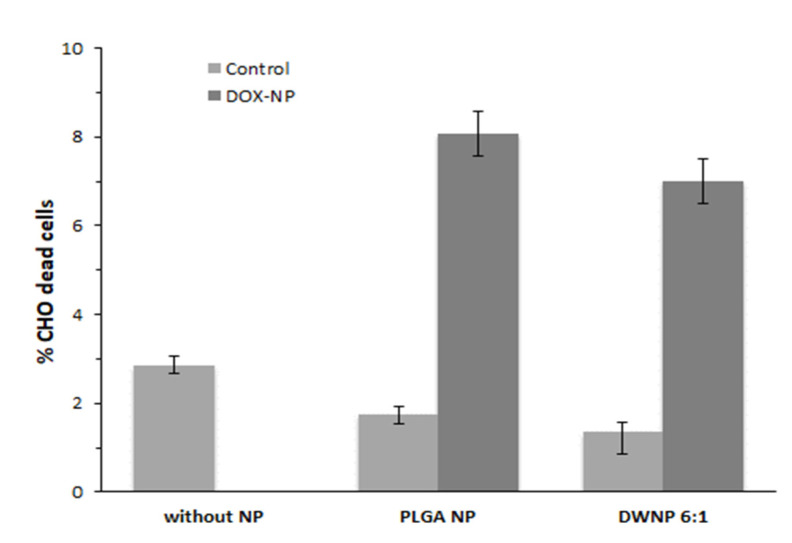
Percentage of dead cells in the presence of 1 mg/mL of unloaded or doxorubicin-loaded PLGA NP and DWNPs 6:1, obtained by flow cytometry, (*n* = 3).

To investigate the in vitro toxicity of the unloaded and DOX-loaded NP produced, mammalian CHO cell lines were exposed to unloaded and DOX-loaded NP for periods of 72 h. Figure 6 and Figure 7 show the effect of DOX-loaded NP on the absolute number of cells (by direct counting), and on the percentages of dead cells (by means of flow cytometry), respectively. As a control, an assay was performed in the absence of NP.

**Figure 8:**
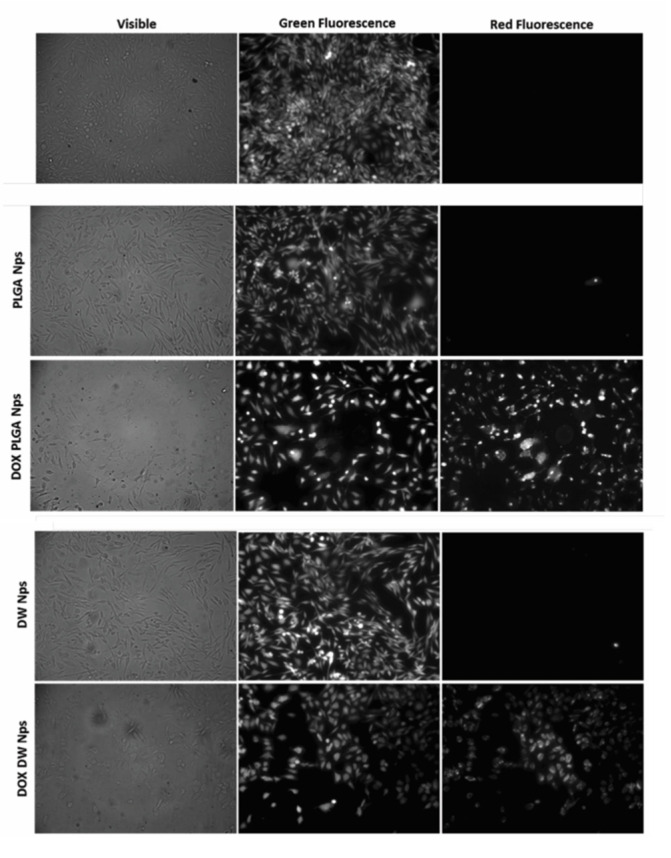
Fluorescence microscopy photographs of CHO cells post-LIVE/DEAD viability/cytotoxicity assay obtained with no addition of NP (negative control) and with the addition of empty and doxorubicin-loaded PLGA NP and DWNPs 6:1. Viable cells fluoresce green while doxorubicin fluoresces red.

Cells count as well as flow cytometric analysis clearly demonstrate that unloaded DWNPs do not affect the number of viable cells exhibiting thereof an excellent biocompatibility. A great increase in the percentage of dead cells is obtained for all DOX-loaded NP confirming that doxorubicin was released. These results were corroborated by the application of the LIVE/DEAD test (Figure 8).

We observed an intense uniform green fluorescence in live cells (due to the presence of calcein) and a reduction in the number of cells grown in monolayer in the experiments performed in the presence of all DOX-loaded NP when compared with the assays performed in the absence of NP or with unloaded NP. No red fluorescence is observed in the experiments with no addition of NP or with the addition of unloaded NP, as expected, while red fluorescence is observed in the experiments with DOX-loaded NP. However, this fluorescence is due to the red color of doxorubicin and not to the presence of dead cells as they are removed by washing procedures (see Experimental). In all cultures grown with the addition of DOX-loaded NP, cells exhibited a round shape which is an indicator of cellular death that occurred previous to detachment.

## 4. Conclusions

DWNPs made of PLGA and PLLA with PLLA/PLGA mass ratios of 2:1, 4:1 and 6:1 loaded with DOX were successfully prepared. The DWNPs produced present a PLGA-rich core where doxorubicin was mainly solubilized surrounded by a PLLA-rich outer phase. The DWNPs produced present very low initial burst values and a sustained DOX release for at least 90 days with release rates decreasing with the increase in the PLLA amount. Zero-order release kinetics were obtained after day 15. These results support that PLLA acts as a rate limiting barrier and strongly indicate that a non-drug loaded outer layer can be effectively used to adjust the drug release rate of DOX from the drug-loaded PLGA core. A setback can be the smaller volume of the core when compared to the single layer PLGA NP which yields lower encapsulation capacity and a concomitant lower concentration of drug released for similar time periods. Nevertheless, the ability to sustain the release for longer periods is promising for applications that require this kind of strategy. The unloaded DWNPs produced were used in in vitro tests with CHO cells and demonstrate that are nontoxic while the DWNPs loaded with the well-known cytotoxic drug DOX caused a great cellular viability decrease when tested in vitro.

## Figures and Tables

**Table 1:** Diffusion exponent n and constant k, according to Equation (1), of doxorubicin release from DWNPs with different PLLA/PLGA mass ratios and PLGA.

Particle	*n*	*n* Error (%)	*k* (day^−1^)	*k* Error (%)	r^2^
DOX–DWNP 2:1	0.58	7.6	3.28	10.6	0.99
DOX–DWNP 4:1	0.49	10.1	2.98	11.3	0.99
DOX–DWNP 6:1	0.43	2.1	1.32	4.9	0.99
PLGA NP	0.62	1.9	6.96	1.5	0.99
